# Combinatorial Pharmacophore Modeling of Multidrug and Toxin Extrusion Transporter 1 Inhibitors: a Theoretical Perspective for Understanding Multiple Inhibitory Mechanisms

**DOI:** 10.1038/srep13684

**Published:** 2015-09-02

**Authors:** Yuan Xu, Xian Liu, Yulan Wang, Nannan Zhou, Jianlong Peng, Likun Gong, Jing Ren, Cheng Luo, Xiaomin Luo, Hualiang Jiang, Kaixian Chen, Mingyue Zheng

**Affiliations:** 1 State Key Laboratory of Drug Research, Shanghai Institute of Materia Medica, Chinese Academy of Sciences, 555 Zuchongzhi Road, Shanghai 201203, China; 2 Center for drug safety evaluation and research, Shanghai Institute of Materia Medica, Chinese Academy of Sciences, 555 Zuchongzhi Road, Shanghai 201203, China; 3 School of Life Science and Technology, Shanghai Tech University, Shanghai 200031, China

## Abstract

A combinatorial pharmacophore (CP) model for Multidrug and toxin extrusion 1 (MATE1/SLC47A1) inhibitors was developed based on a data set including 881 compounds. The CP model comprises four individual pharmacophore hypotheses, HHR1, DRR, HHR2 and AAAP, which can successfully identify the MATE1 inhibitors with an overall accuracy around 75%. The model emphasizes the importance of aromatic ring and hydrophobicity as two important structural determinants for MATE1 inhibition. Compared with the pharmacophore model of Organic Cation Transporter 2 (OCT2/ SLC22A2), a functional related transporter of MATE1, the hypotheses of AAAP and PRR5 are suggested to be responsible for their ligand selectivity, while HHR a common recognition pattern for their dual inhibition. A series of analysis including molecular sizes of inhibitors matching different hypotheses, matching of representative MATE1 inhibitors and molecular docking indicated that the small inhibitors matching HHR1 and DRR involve in competitive inhibition, while the relatively large inhibitors matching AAAP are responsible for the noncompetitive inhibition by locking the conformation changing of MATE1. In light of the results, a hypothetical model for inhibiting transporting mediated by MATE1 was proposed.

In the past decades, numerous studies have suggested that transporters in human play a significant role in pharmacokinetic processes including drug absorption, disposition and elimination. With the accumulation of knowledge about transporters, Food and Drug Agency (FDA) has specified a few major transporters that mediated clinical significant drug-drug interactions (DDIs) (http://www.fda.gov/Drugs/DevelopmentApprovalProcess/DevelopmentResources/DrugInteractionsLabeling/ucm080499.htm), including: P-gp, organic anion transporting polypeptides (OATPs), breast cancer resistance protein (BCRP) and organic anion transporter (OAT). Decision trees for P-gp, BCRP, OATP, OCT (organic cation transporter) and OAT were proposed as guidelines to decide whether a new chemical entity (NCE) needs an *in vivo* DDI study^1^. Since it is poorly understood, the mammalian multidrug and toxin extrusion (MATE) transporter has attracted more and more attention because of its clinical importance.

MATE1 was first identified in 2005^2^, which is a twelve transmembrane efflux transporter, encoded by SLC47A1 gene. MATE1 is widely distributed in body tissues, including the kidney, liver, skeletal muscle, adrenal gland, and testis^3^. In the kidney, MATE1 is localized to brush-border membrane of proximal tubules, which is a key player in renal excretion process. After the uptake by basolateral membrane transporter OCT2, many exogenous and endogenous substances can be subsequently pumped out from renal cell into urine by MATE1, driven by an outward H^+^ gradient. Therefore, it is significant to understand the MATE1-mediating transporting, which may help to elucidate the tissue distribution and excretion process of drugs. Typical cationic drugs like metformin and cimetidine are substrates of MATE1^4^. MATE1 also transport anionic compounds, such as acyclovir and ganciclovir^5^. Obviously, MATE1 inhibition may result in increased substrate concentrations in the renal tubule, which is often accompanied by drug adverse side effects. It was reported that plasma concentration and renal accumulation of cisplatin are higher in the MATE1 knock-out mice^6^. Furthermore, compared with the use of cisplatin alone, the combined use of a selective MATE1 inhibitor with cisplatin also elevated the creatinine concentration in mice, which suggested that abnormal function of MATE1 may be involved in cisplatin-induced nephrotoxicity. A systematic analysis of the inhibition potency of cimetidine for the influx and efflux transporters of organic cations suggested that the inhibition of MATEs, instead of OCTs, should be the mechanism underlying the related DDIs^7^. These results emphasize that a better understanding about the transporting mechanisms of MATE1 in renal clearance is of particular relevance to predicting and avoiding unwanted DDIs.

Despite the predominant role in renal secretion, there are relatively limited studies to comprehensively explore the structural patterns of MATE1 ligands. Astorga *et al.* determined the IC_50_ values of 59 structurally diverse compounds by measuring the uptake of the substrate 1-methyl-4-phenylpyridinium (MPP^+^) for both hMATE1 and hMATE2-k^8^. In addition, a quantitative pharmacophore and a Bayesian model for MATE1 inhibitors were developed based on the investigated compounds, highlighting some molecular fragments and structural features favoring the interaction of inhibitors with MATE1. Recently, 898 prescription drugs were screened with ASP^+^ (4-(4-(dimethylamino)styryl)-N-methylpyridinium iodide) as the substrate probe, and 84 potential MATE1 inhibitors were found^9^. Different computational models were constructed for predicting MATE1 inhibitors based on the data, among which the random forest model showed the highest prediction performance with an average AUC 0.78 ± 0.02. The resulted model was used to perform an *in silico* screening against a large compound library, and successfully identified four hits that were later verified by *in vitro* assays. Both the studies indicated that MATE1 inhibitors are markedly overlapped with the inhibitors of other organic cation transporters like MATE2-k and OCT2. Although the possible mechanisms for the inhibition remain unclear, these studies provide valuable information for our understanding their interaction with ligands.

Compared to the drug targets such as enzymes or receptors, membrane transporters are characterized by their low binding affinity and high structural promiscuity of ligands, which means that multiple mechanisms may be involved in the ligand binding and transporting process. Accordingly, such systems may require some problem-oriented strategies. In our previous study, we designed a combinatorial pharmacophore (CP) modeling protocol to investigate the multiple inhibitory mechanisms of OCT2^10^. Generally, a pharmacophore defines the spatial arrangements of molecular features that are necessary to ensure the interactions with a specific biological target. For membrane transporters, since a combination of many different binding modes can be involved in their interaction with ligands, we tried to identify the optimum pharmacophore combination by an exhaustive searching algorithm.

In this study, we employed the CP modeling strategy to study the interaction between MATE1 and its ligands. By combining the matching results of different pharmacophore hypotheses, the CP model can not only discriminate inhibitors from noninhibitors, but also differentiating inhibitors acting via varied mechanisms. It is assumed that each pharmacophore hypothesis in CP model represents a particular interaction pattern between inhibitors and MATE1. Accordingly, a ligand will be defined as an inhibitor once it matches any one or more constituent hypotheses. By systematically searching from a total of 3,422 pharmacophore hypotheses and 20,475 candidate combination models, the final CP model consisting of four individual hypotheses, HHR1, DRR HHR2 and AAAP, yielded reasonable performance on discriminating a total of 881 MATE1 inhibitors and noninhibitors. To further analyze how the different pharmacophore hypotheses are associated with different inhibitory mechanisms, the homology model of hMATE1 was constructed, and molecular docking was performed to investigate the binding modes of inhibitors matching a particular pharmacophore hypothesis. Additionally, the different pharmacophore features of MATE1 and OCT2 inhibitors are compared.

## Results and Discussion

### Performance comparison of individual and combinatorial pharmacophore modeling

**Figure 1:**
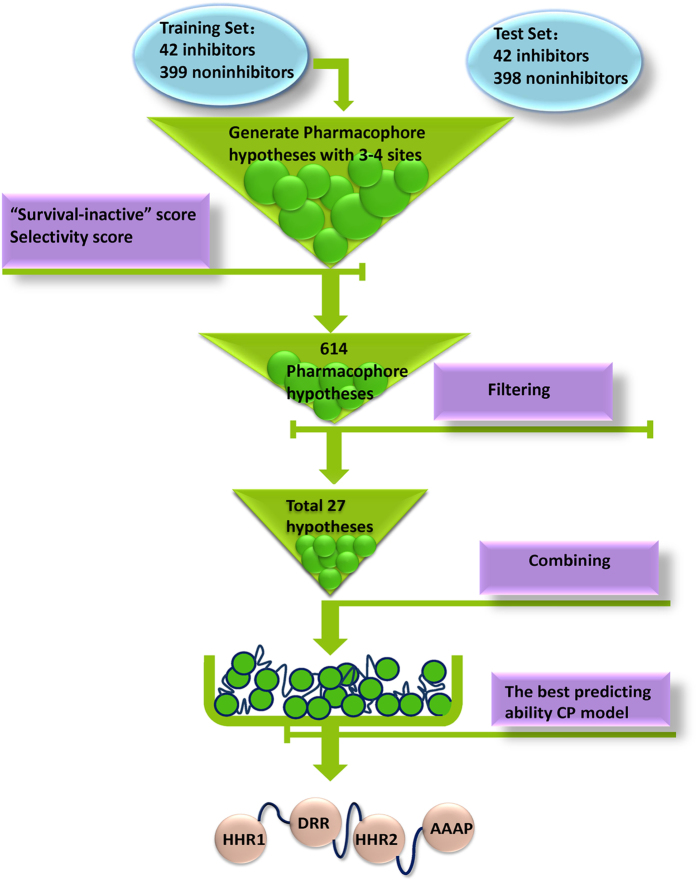
Work flow for developing combinatorial pharmacophore model. The criteria used to develop CP model and the number of hypotheses kept in each step are noted. The final CP model with the best prediction performance is composed of HHR1, DRR, HHR2 and AAAP.

The work flow for developing the CP model is showed in Fig. 1. A total of 3,422 pharmacophore hypotheses with 3 or 4 sites were created based on 42 inhibitors in the calibration set. Then, 614 hypotheses were retained after the filtering of “Survival-inactive” score and selectivity score, among which 27 hypotheses that matched by fewer noninhibitors were selected. These 27 hypotheses (Supplementary Table S1) were combined to yield a complete enumeration of all possible combinations of any three or four hypotheses and altogether 20,475 candidate CP models were developed. The CP model with the highest value of balanced-accuracy (BACC) was finally selected for subsequent study.

Due to the skewness of our data, the accuracies (ACCs) of single pharmacophore hypotheses can be highly inflated by the large number inactive prediction, not reflecting the true prediction capability. We noticed that sensitivities (SEs) of all single pharmacophore hypotheses are low, ranging 0.19 to 0.26, which suggests that none of them can fully explain the binding of MATE1 inhibitors. The CP model, instead, can successfully discriminate the inhibitors and noninhibitors in the calibration set and test set with the accuracy of 0.76 and 0.73, respectively. Compared to any single phamacophore hypotheses, the SE of CP model was dramatically enhanced (Table 1). It means that the combination procedure effectively increases the capability of pharmacophore model in recognizing the “true” MATE1 inhibitors, which is significant for improving our understanding about MATE1 inhibition mechanisms.

### Component analysis of the CP model of MATE1 inhibitors

**Figure 2:**
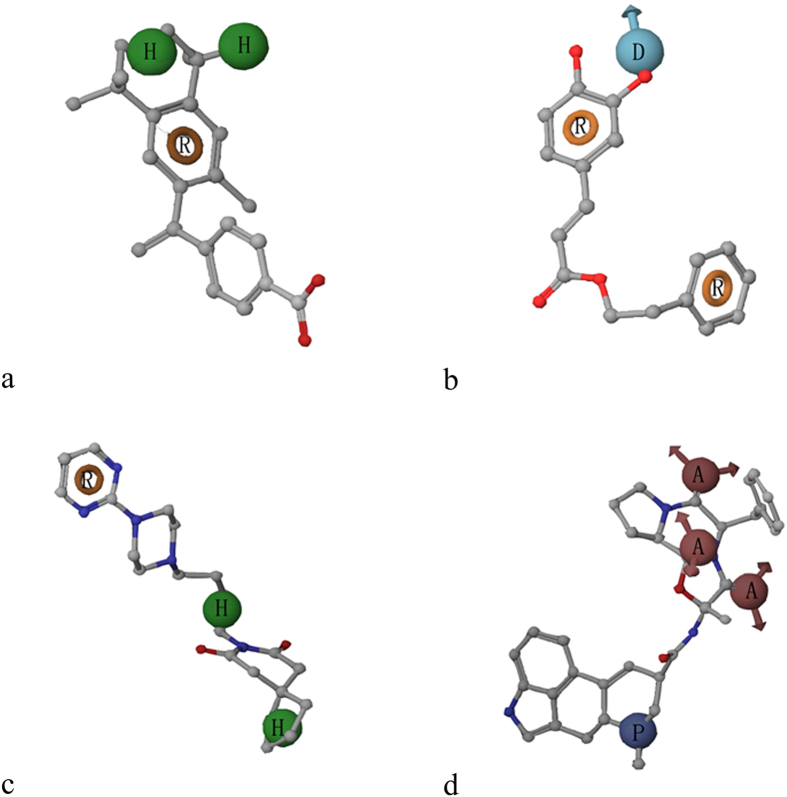
Illustration of the pharmacophore hypotheses in the final CP model matched by different MATE1 inhibitors: (**a)** HHR1 with bexarotene. (**b)** DRR with caffeic acid phenethyl ester. (**c**) HHR2 with buspirone. (**d**) AAAP with ergotamine. Pharmacophore features in each hypothesis are labeled, where A represents hydrogen bond acceptor, D represents hydrogen bond donor, H represents hydrophobic feature, P represents positive charge, and R represents aromatic ring.

To interpret the role of the four pharmacophores in MATE1 inhibition, a series of detailed analysis on these hypotheses were carried on, covering the aspects of the pharmacophore features in the CP model, molecular weights of inhibitors matching each pharmacophore hypothesis and representative compounds. As shown in Fig. 2, the component hypotheses in CP model included HHR1, DRR, HHR2 and AAAP. The site spatial measurements of every hypothesis including site distances and angles are listed in Supplementary Table S2 and Table S3. Among the pharmacophore features, aromatic rings are required by three hypotheses, and hydrophobic center is another common feature in the CP model, implying that these two interaction patterns are crucial for the recognition between MATE1 and its inhibitors. Positive charge, which is quite important in OCT2 inhibition^10^, is only present in one pharmacophore AAAP. A previous research reported HHAP as an important pharmacophore pattern of MATE inhibitors^8^, while the data used for modeling was measured with MPP^+^ as the substrate probe. This result may suggest that the mechanism of transport inhibition is substrate-dependent, supporting the concept that the binding of substrates to MATE transporters involves interaction with a binding “surface” containing multiple binding “sites”^11^. In the end, both pharmocophore models of MATE1 inhibitors highlighted the vital role of the hydrophobic interaction.

**Figure 3:**
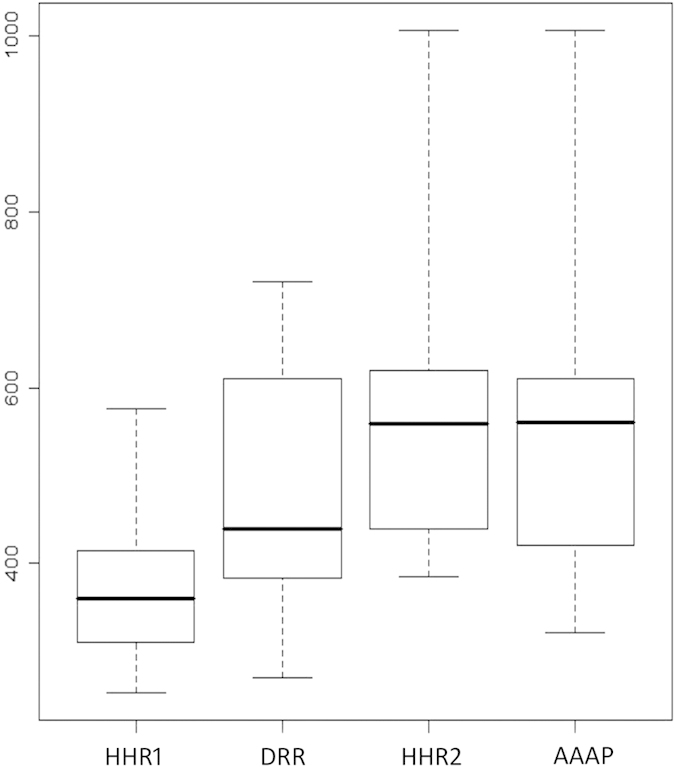
Boxplot of molecular weights of inhibitors matched different pharmacophore hypotheses.

To investigate the specific patterns underlying each pharmacophore hypothesis, molecular weight (MW) of different types of inhibitors were compared. Overall, MATE1 inhibitors were slightly larger than OCT2 inhibitors, and more MATE1 inhibitors appeared in the range from 500–750 Da (Supplementary Figure S1). Among those four pharmacophore hypotheses, the average MW of HHR1 matched inhibitors was the smallest (Fig. 3), with the median of 360.36 Da and the maximum of 575.68 Da (Table 2). Most inhibitors matching HHR2 or AAAP are large in size with the median over 550 Da (Table 2). Some inhibitors with over 1000 Da can even be allowed by HHR2 and AAAP. Inhibitors matching DRR cover a wide range of molecular weights, in which the majority of inhibitors are relative small with the median of 438.69 Da, but large inhibitors with 720 Da are also allowed. Generally, the inhibitors matching HHR1 are relatively small, but the inhibitors matching HHR2 or AAAP are much larger.

To examine the relationship between each pharmacophore hypothesis and different mechanisms of inhibition, some known MATE1 inhibitors or substrates were analyzed. As there were no compounds that were directly reported as competitive inhibitors for ASP^+^, “potential competitive inhibitors” were analyzed, which was identified if it had been reported as an inhibitor for ASP^+^ and a substrate for MATE1 as well. Among the previously reported substrates, only cimetedine and topotecan were also identified as hMATE1 inhibitors in Wittwer’s experiments^9^. In our CP model, cimetedine was found matching HHR1 and topotecan matching DRR. Interestingly, the substrate probe in our set, ASP^+^, was also found matching HHR1 and DRR. The common pharmacophore of HHR1 or DRR among cimetedine, topotecan and the substrate probe ASP^+^ indicated that these three ligands may bind to the same site of MATE1, and two pharmocophore hypotheses HHR1 and DRR should involve in competitive inhibition.

The substrates of transporters typically bind to a relatively small pocket in a big transporting chamber^12^,^13^. Based on above analysis of molecular weights of each hypothesis, the inhibitors matching HHR1 and DRR are indeed smaller in size, allowing them to get into the substrate binding site and competitively inhibit the transporting of ASP^+^. In addition to competitive binding the same site with substrate, transporter inhibitors can also act in a noncompetitive manner through locating in transporting chamber, occluding the substrate binding site and locking the conformation transformation of transporter^12^,^13^. For example, MaD5, MaD3S and MaL6 are large peptides that can inhibit the transportation of EtBr by pfMATE^12^. Previous crystallographic studies have revealed that the substrate is located in the N-lobe region of pfMATE, while MaL6 bind at the central lobe due to a relatively larger molecular size, which may affect the transporting of substrate in a noncompetitive manner. In contrast, the peptide residues in the mini cycle head of MaD5 and MaD3S filled the N-lobe cavity, and may hence directly affect substrate binding. Here, these three inhibitors of pfMATE, MaD5, MaD3S and MaL6, were also searched with our CP model of hMATE1.This result showed that only large MaL6 can match AAAP, while MaD5 can match DRR, and MaD3S can match DRR and HHR2. From the molecular weight analysis shown in Fig. 3, we may notice that the inhibitors matching AAAP are relatively large among all hMATE1 inhibitors, implying that interaction site of phamacophore AAAP are located in the larger central chamber but not inside the small substrate binding site. Overall, these results indicate that HHR1 and DRR correspond to the interaction model for small inhibitors that bind into the N-lobe, while AAAP is related to the model for relatively large molecules that bind to the central chamber.

### Comparison of the CP models between MATE1 and OCT2

In the kidney, OCT2 and MATE1 have been shown to act concertedly in the excretion of organic cations that OCT2 firstly uptakes drugs into renal cells and MATE1 subsequently pumps drugs out of the cells. Their substrates and inhibitors specificity are supposed to partially overlap^14^. However, some compounds selectively inhibit one of these two types of transporters. Understanding this selectivity can help us answer how important different structural features are for a specific human cation transporter and for the distribution and elimination of drugs. Here, we compared pharmacophore features in each CP models of MATE1 inhibitors with those of OCT2 inhibitors. For OCT2, three of four hypotheses in the final CP model (DHPR18, APR2, PRR5 and HHR4) included the positive charge feature^10^. For MATE1, the positive charge only appeared in one of the hypotheses, suggesting that the positive charge feature for MATE1 inhibitors is not as important as for OCT2 inhibitors.

**Figure 4:**
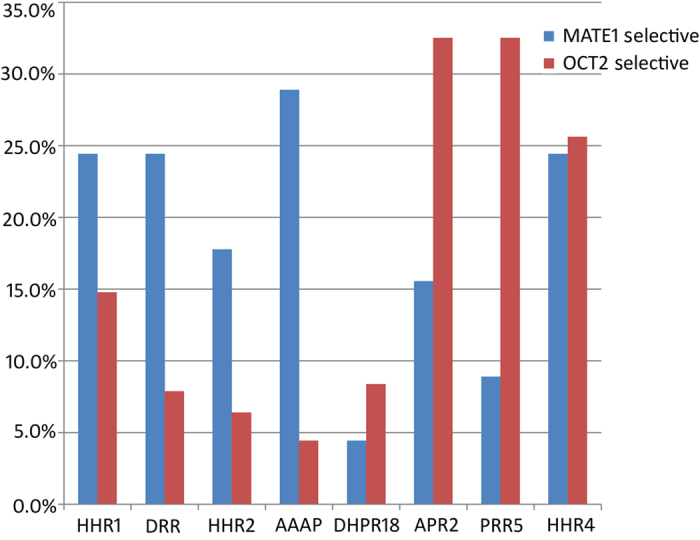
Histogram to compare the distribution of MATE1 selective, OCT2 selective and dual inhibitors in matching hypotheses. MATE1 selective inhibitors and OCT2 selective inhibitors are colored by blue and red, respectively. Bars show the percentage of particular inhibitors matching each pharmacophore hypothesis.

In our data set, there are 45 MATE1 selective inhibitors, 203 OCT2 selective inhibitors and 39 dual inhibitors. In order to understand which pharmacophore features differentiate three types of inhibitors, the matching pharmacophore hypotheses of three types of inhibitors were further exploited. Pharmacophore hypotheses from MATE1 CP model and OCT2 CP model were searched by OCT2 selective and MATE1 selective inhibitors, respectively. Figure 4 shows the differences of these different inhibitors on matching altogether eight pharmacophore hypotheses from both MATE1 and OCT2 CP models. It can be found that AAAP can better discriminate between OCT2 and MATE1 selective inhibitors, where a very small number of OCT2 selective inhibitors and a large number of MATE1 selective inhibitors can match the pharmacophore. Wittwer *et al.* reported that the range of molecular weight and volume of MATE1 inhibitors were much wider than OCT2 inhibitors^9^, which means that MATE1 can tolerate much larger ligands than OCT2. Therefore, the MATE1selective inhibition represented by AAAP suggest that MATE1 should have a more spacious transporting cavity as compared to OCT2. For OCT2 selective inhibition, nearly equal amount of OCT2 selective inhibitors match APR2 and PRR5, respectively, but much fewer MATE1 selective inhibitors can match PRR5. Our previous study has revealed that PRR5 is involved in the competitive inhibition of ASP^+^ in OCT2^10^. The current study suggests that PRR5 is also important for its OCT2 selectivity over MATE1. Remarkably, both models emphasized the role of two hydrophobic and one ring. Among three HHR hypotheses, HHR1 derived for MATE1 and HHR4 derived for OCT2 show similar structural arrangement (Supplementary Table 2 and Supplementary Table 4), and these two pharmacophores can be matched by more than half of dual MATE1 and OCT2 inhibitors. These results suggested that HHR1 and HHR4 are the common interaction pattern shared by both MATE1 and OCT2. Once the molecule matched HHR1 or HHR4, it is likely to be a dual inhibitor of MATE1 and OCT2.

### Molecular docking analysis of the compounds with different pharmacophore matching

**Figure 5:**
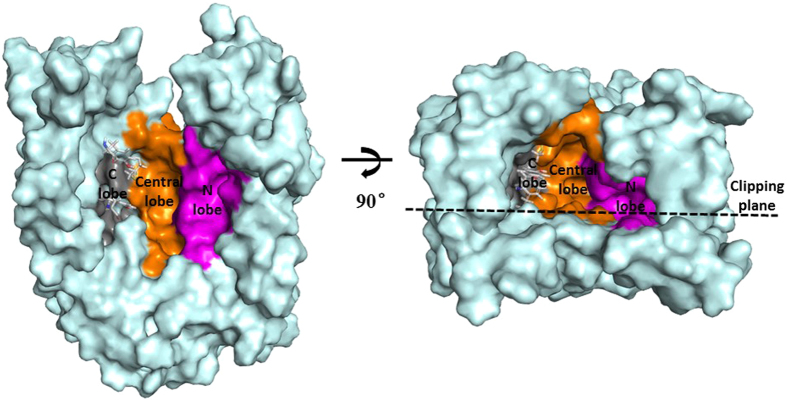
The structure of transporting chamber. C lobe, central lobe and N lobe are colored in grey, orange, and magenta, respectively. The residues that occupied the binding space of C lobe are displayed in sticks.

The above analyses revealed that different pharmacophores are related to different binding patterns of MATE1 inhibitors. It is therefore interesting to investigate the putative binding modes of these inhibitors within the structure of hMATE1. Sequence alignment of hMATE1 and two template structures NorM-VC and pfMATE was provided in Supplementary Figure S2. Three homology models of hMATE1 constructed basing on the template of pfMATE, NorM-VC and both these two structures were compared. Among them, the model based on the pfMATE got the highest QMEAN6 score of 0.484 (Table 3). The PROCHEK result of the model based on the pfMATE show that the model is also the one showing the most reasonable stereo chemical features, where 99.5% of residues have been found in the favored regions (Supplementary Figure S3b), suggesting the high quality of the resulted model. This model was therefore selected for the followed analyses. As shown in Supplementary Figure S3a, all twelve transmembrane domains (TMD) were successfully constructed and the model can be well aligned to the structure of pfMATE. In the homology model structure, two bundles of six transmembrane helices, N-terminal half (TMD1–6) and C-terminal half (TMD7–12), formed a large and outward-opening transporting chamber. Particularly, the transporting chamber can be divided into an N lobe, a central lobe and a C lobe cavity (Fig. 5), among which the N lobe is smaller than the central lobe and located deeper in the chamber. The central lobe cavity includes the rest of transporting chamber from the entrance to the bottom of the transporting pathway. In addition, C lobe cavity is much smaller than N lobe, and the binding space in C lobe is occupied by the non-conservative residuals Met272, Gln295 and Tyr299 on TMD7 and TMD8. This result is consistence with the crystal structure of pfMATE, where the residues located at the boundary of C lobe and central lobe and would interfere the entrance of C lobe^12^. Therefore, there are mainly two substrate binding sites in hMATE1, as revealed from our homology modeling structure.

**Figure 6:**
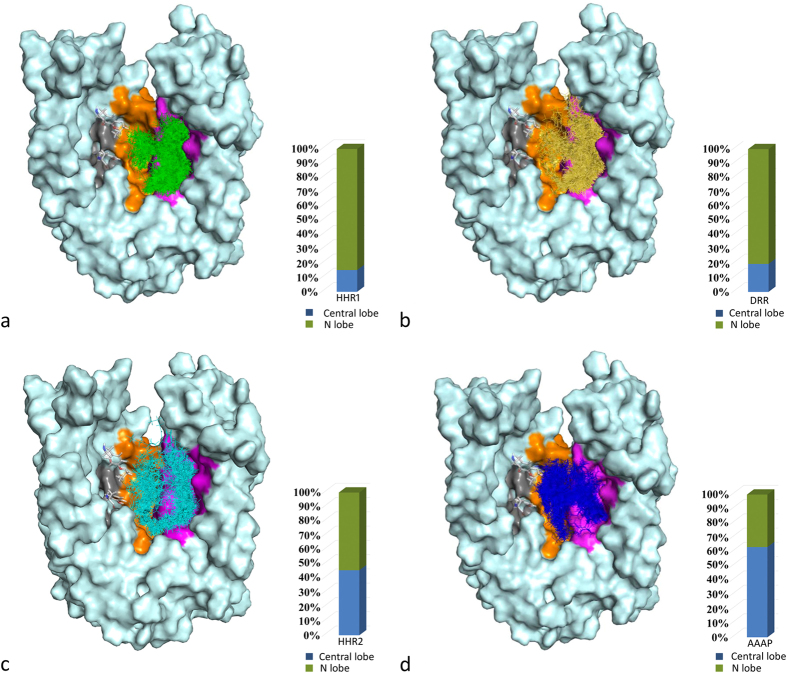
Putative binding poses of inhibitors matching each pharmacophore hypothesis. Bars show the distribution of binding poses located in different regions of MATE1 binding site.

The low energy binding modes of inhibitor matching different pharmacophore hypotheses were generated by flexible docking them into the constructed MATE1 structure. The distributions of these binding modes were classified into two types based on their geometric center locations: (1) within N lobe cavity or (2) within the central cavity. The inhibitors only matching HHR1, DRR, HHR2 or AAAP, were firstly analyzed. As shown in Fig. 6a,b, docking results revealed that the inhibitors matching HHR1 or DRR have a similar overall binding pose distribution which are majorly located in N lobe. While, the inhibitors matching AAAP (Fig. 6d) show a different binding pose distribution, which are majorly located in the central lobe. Interestingly, the inhibitors matching HHR2 (Fig. 6c) can bind to both N lobe and the central lobe, of which the binding poses resemble a “U” shape thread running through the whole binding chamber. As shown in Fig. 6a, 84.78% binding modes of the inhibitors matching HHR1 and 80.44% binding modes of DRR are located in N lobe cavity. In contrast, for the inhibitors matching AAAP, only 36.79% of its binding modes are located in N lobe, and all the others present in the central cavity. The inhibitors matching HHR2, just as noticed in Fig. 6c, have nearly equal distribution of their binding poses within these two binding sites (54.49% in N lobe and 45.51% in central cavity). Clearly, these results support our previous argument that HHR1 or DRR should correspond to the ligand binding model within the small N lobe cavity, and AAAP the more spacious central cavity. However, due to the limited available data (the inhibitors only matching HHR2 were analyzed as mentioned before), there is insufficient evidence to support any of assumptions for HHR2. More research is needed to decide the ligand binding model of HHR2.

**Figure 7:**
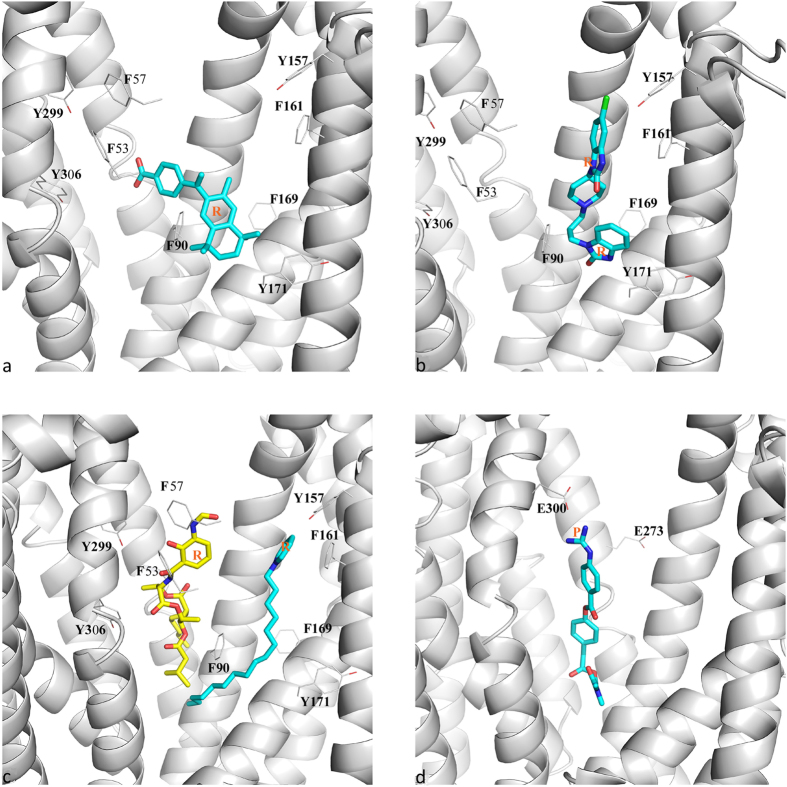
Representative binding modes of ligands matching each pharmacophore hypothesis. The ligands are in the docked conformations that can match corresponding pharmacophore hypotheses. The pharmacophore feature of ring and positive charge are labeled as R and P, respectively. (**a)** The binding mode of the inhibitor matching HHR1. (**b)** The binding mode of the inhibitor matching DRR. (**c)** Two different binding modes of inhibitors matching HHR2. (**d**) The binding mode of the inhibitor matching AAAP. Figures were produced using PyMOL (DeLano Scientific LLC, Palo Alto, CA, USA. http://www.pymol.org).

A few representative docking conformations were also extracted to explore the mapping between the corresponding pharmacophore features and their adjacent residues. For example, the ring feature of HHR1 (Fig. 7a) is close to Phe90 located at lower part of TMD2, which is partially overlapped with one of ring features of DRR (Fig. 7b). Another ring feature of DRR can extend to the upper part of TMD4 around Tyr157 and Phe161. For the ring feature of HHR2, the mapped ligand moieties may be either located in N lobe or the central lobe. However, all the binding regions are surrounded by multiple ring-containing residues such as phenylalanine and tyrosine (Fig. 7c). These observations suggested that aromatic residues are important for the ligand binding of hMATE1, which may involve in extensive π-π interaction with the ring features of inhibitors represented by HHR1, HHR2 and DRR. The charged residues are also crucial for inhibitors recognition which may form electrostatic interaction. The docking conformation of AAAP indicated that the feature of positive charge is near to Glu300 on TMD8 (Fig. 7d), which is an essential residue for maintaining the activity of MATE1^15^.

### Hypothetic models for substrate transporting of MATE1 and inhibition mechanisms

MATE1 is a key element for pharmacokinetics, pharmacodynamics and toxico dynamics which completed the renal elimination process of organic cations and was considered as one of the sites of DDIs^16^,^17^. Relatively little attention has been given to determine the structural basis of inhibitor interaction with MATE transporters. Considering the wide range of inhibitors and the complex mechanisms involving in inhibition, we developed the combinatorial pharmacophore model of MATE1 inhibitor of which each hypothesis represented one pattern of inhibition, with the largest data set of MATE1 inhibition to date. The CP model is to provide useful information about MATE1 inhibitory mechanisms.

**Figure 8:**
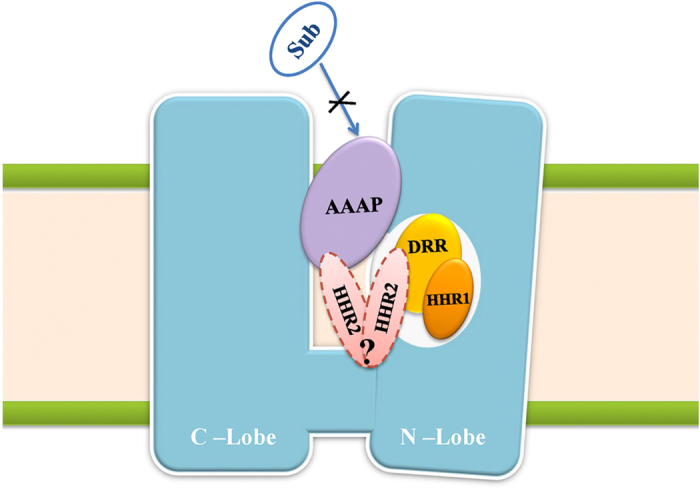
Hypothetic models for MATE1 inhibition. Sub: substrate. Inhibitors matching HHR1 or DRR compete for substrate binding site and competitively inhibit the transporting of the substrate. Inhibitors matching AAAP lock the transporter conformation transformation and noncompetitively inhibit the transporting of the substrate. The binding site of inhibitors matching HHR2 can be in either the N lobe or the central lobe.

The analysis of molecular weight and representative inhibitors implied that HHR1 and DRR may involve in competitive inhibition, while AAAP may be responsible for noncompetitive inhibition. Docking result further supported our inference. Therefore, hypothetic models for hMATE1 inhibition are concluded that small inhibitors matching HHR1 and DRR can get into the transporting chamber, interact with MATE1 at relatively small substrate binding site located in inner chamber, and affect transportation by competing the binding site with the substrate. On the other hand, inhibitors matching AAAP which are usually large compounds could not be tolerated by the small substrate binding site but located in large central cavity, which may noncompetitively inhibited the MATE1 by locking the conformation changing (Fig. 8). Due to the small number of inhibitors which only match HHR2, it was still uncertain which kind of inhibition HHR2 involved in or by both in competitive and noncompetitive manner that HHR2 can affect MATE1 as the sites distribution almost same in N lobe and central lobe. Other noncompetitive inhibition that affected H^+^ binding or allosteric regulation may participate in the transporting process. Lacking of the specific experimental data, our model at present couldn’t cover these noncompetitive pattern.

## Conclusion

MATE1 is the critical player in the renal elimination which is responsible for transporting drugs from renal cell to urine. Substantial evidence has accumulated implicating that MATE1 have a profound effect on pharmacokinetic properties of drugs and DDIs. To gain basic knowledge and help develop drug with a favorable safety profile, it is of great interest to investigate what structural factors and how structural factors affect MATE1 transporting process. Here, we developed a combinatorial pharmacophore (CP) model for MATE1 inhibitors, consisting of HHR1, DRR, HHR2 and AAAP. The CP model not only well discriminated inhibitors and noninhibitors, but also identified hydrophobic and aromatic ring features that are important for MATE1 inhibition. These results may help us to gain insights into the mechanisms of MATE1 inhibition, to identify MATE1 inhibitors in the early phase of drug discovery, and to avoid unwanted DDIs. All analysis about molecular weight, representative inhibitors and docking pattern of four hypotheses suggested that HHR1 and DRR are related to the ligand binding model within N lobe, of which the mapped ligands may inhibit transporting by a competitive manner. While large inhibitors matching AAAP are more likely to be located in the central cavity and noncompetitively inhibit transporting process by locking the conformation changing. These results may pave the way towards better understanding of MATE1 transporting mechanisms.

## Methods

### Data Set

We collected a total of 881 compounds from the literature^9^, among which 84 ligands are inhibitors of MATE1. Only the compounds which have been screened for OCT2 inhibition^18^ and used to generate OCT2 CP model^10^ were obtained from the literature. These compounds were randomly divided into a calibration set with 42 inhibitors and 399 noninhibitors, and a test set with 42 inhibitors and 398 noninhibitors. The chemical structures of these compounds were downloaded from PubChem website. The LigPrep module (LigPrep, version 2.3, Schrödinger, LLC, New York, NY) was used to produce low-energy three-dimensional (3D) conformations for the compounds using OPLS-2005 force field. The chirality centers were kept during the process and the tautomerization and ionization states were adjusted at a pH of 7.4. A maximum 1000 of conformations per structure were generated.

### Generate Combinatorial Phamacophore model

Firstly, 42 inhibitors in calibration set were used to generate pharmacophore hypotheses with the Phase^19^ (Phase, version 3.2, Schrödinger, LLC, New York, NY). Then, six pharmacophore features, including hydrogen bond acceptor (A), donor (D), hydrophobic (H), negative charge (N), positive charge (P), and aromatic ring (R), were searched and defined for each structure. Based on a binary decision tree algorithm, the Phase module was used to find common pharmacophore consisting 3 to 4 sites. Secondly, “Survival-inactive” score and Selectivity score were used to rank and select the generated individual pharmacophore hypotheses prior to the CP modeling. Here the term “survival” means a measure of how well a hypothesis can be matched by inhibitors in terms of their spatial overlap, and the “Survival-inactive” score can not only take into account of the extent of matching by inhibitors, but also impose penalty on the matching of noninhibitors. Selectivity score is an empirical estimate of the rarity of a hypothesis, which measures what fraction of molecules is likely to match the hypothesis, regardless of their activity toward the receptor. A higher Selectivity score means that the hypothesis is more likely to be unique to the inhibitors, which is useful to control the false positive rate of compound matching. Thirdly, all the selected hypotheses were combined into N-member groups in all possible manners, where N can be 3 and 4 to account for multiple binding modes of different MATE1 inhibitors. Each combinatorial pattern corresponds to a potential CP model. The sensitivity (SE), specificity (SP), accuracy (ACC) and balanced-accuracy (BACC) were calculated to assess each model, as defined below:


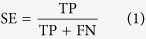



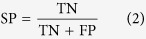



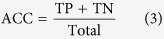



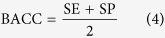


where TP is true positive, TN is true negative, FP is false positive, FN is false negative prediction, and the sum of them is the number of all the compounds in the calibration or test set. In this study, a TP means that a MATE1 inhibitor is successfully matched by any of the constituent hypotheses of a candidate CP model. As the data set is highly skewed (i.e. the number MATE1 inhibitors is significantly lower than noninhibitors), we use BACC here to balance between SE and SP, and a higher BACC means a better performance on discriminating inhibitors and noninhibitors based on an overall consideration. In the end, the CP model with the highest BACC was selected for further investigation.

### Develop the homology model of hMATE1

The FASTA sequence of human MATE1 was retrieved from NCBI protein sequence database (Accession: Q96FL8). Four steps were followed to develop the homology model of hMATE1. (1) Templates selection. The BLAST^20^ program was used to search suitable template available in the PDB. Two crystal structures of NorM from *Vibrio cholera*^21^ (NorM-VC, 3MKT) with a resolution of 3.65 Å and a MATE multidrug transporter from *P. furiosuswere*^12^ (pfMATE, 3VVN) with a resolution of 2.40 Å were obtained, and both of them shared 24% sequence identity with hMATE1. (2) Sequence alignment. Sequences of hMATE1, NorM-VC and pfMATE were aligned by the “align2d” command in MODELLER 9.13^22^, and then manually adjusted according to the work of Tanaka *et al.*^12^. (3) Model Generation. Three types of homology models were developed by Modeller, in which two of them were built from single template alignments with NorM-VC and pfMATE, respectively, and the other was built from a multiple sequence alignment with both two proteins as templates. For each of them, five individual models were generated. The DOPE score was used for selecting one candidate model for each type of constructed models, where native-like models tend to yield lower DOPE scores. (4) Models Validation. “QMEANscore6”, in SWISS-MODEL workspace^23^ was used for further assessment to select the final model, which is a composite scoring function describing the major geometrical aspects of protein structures to identify the native structure or high quality models^24^. Additionally, the models were also validated by PROCHECK analysis^25^.

### Ligands Docking

Inhibitors matched by different pharmacophore hypotheses were docked into the selected hMATE1 homology model with Glide^26^ (Glide, version 5.6, Schrödinger, LLC, New York, NY). The preparation of the protein was done using Protein preparation wizard tool in the Maestro (Maestro, version 9.0, Schrödinger, LLC, New York, NY). This process fixed the protein structure by verifying proper assignment of bonds and bond orders, adding hydrogens, and deleting unwanted bound water molecules. Further, the protein structure was subjected to restrained minimization using the OPLS2005 force field. All the parameters in this module were used with default values unless noted. We used the interaction patter of the substrate, norfloxacin-derivative compound (Br-NRF), and pfMATE protein as the reference to define the active site of hMATE1. A large (14 Å × 14 Å × 14 Å) grid box encompassing the entire substrate transporting chamber was established to take account of all potential binding sites of inhibitors. Besides, the value of “the length of Dock ligands” was also set as the largest value of 36 Å to allow for inhibitors with large size. The ligands were docked using “standard precision” (SP) mode and the protein was set as rigid. The docked poses were evaluated using Glide Score and 100 poses per ligand were written out. Finally, top ranked 30 binding poses (in terms of Glide score) of each ligand were remained for further analysis. In order to identify the interaction pattern of hMATE1 and inhibitors matching different pharmacophore, the relative locations of different inhibitors in the pocket of hMATE1 were analyzed based on the geometric centers of the ligand structures.

## Additional Information

**How to cite this article**: Xu, Y. *et al.* Combinatorial Pharmacophore Modeling of Multidrug and Toxin Extrusion Transporter 1 Inhibitors: a Theoretical Perspective for Understanding Multiple Inhibitory Mechanisms. *Sci. Rep.* **5**, 13684; doi: 10.1038/srep13684 (2015).

## Supplementary Material

Supplementary Information

## Figures and Tables

**Table 1:** Comparison of the prediction performance of CP model and the single pharmacophore hypotheses.

Model	Calibration set				Test set			
	SE	SP	ACC	BACC	SE	SP	ACC	BACC
HHR1	0.24	0.88	0.82	0.56	0.29	0.85	0.80	0.57
DRR	0.26	0.92	0.85	0.59	0.26	0.91	0.85	0.59
HHR2	0.19	0.96	0.89	0.58	0.17	0.95	0.87	0.56
AAAP	0.26	0.94	0.88	0.60	0.17	0.94	0.87	0.56
CP model	0.74	0.76	0.76	0.75	0.57	0.74	0.73	0.66

**Table 2:** The average and range of molecular weights (MW) for each pharmacophore hypothesis.

Model MW	HHR1	DRR	HHR2	AAAP
Average (Mean ± SD)	372.58 ± 85.75	474.89 ± 135.96	568.05 ± 162.15	546.66 ± 161.03
Median	360.36	438.69	559.78	560.73
Min	252.34	270.24	384.44	321.37
Max	575.68	720.94	1007.19	1007.19

**Table 3:** The evaluation of each models based different template(s).

Template	QMEAN6	Ramachandrams Plot			
	score	Core%	Allow%	Gener%	Disall%
pfMATE	0.484	91.0	7.5	1.0	0.5
NorM-VC	0.414	85.5	11.8	2.0	1.3
pfMATE&NorM-VC	0.457	89.7	9.0	0.8	0.5
