# Supplementary material for: Combinatorial Pharmacophore Modeling of Multidrug and Toxin Extrusion Transporter 1 Inhibitors: a Theoretical Perspective for Understanding Multiple Inhibitory Mechanisms
Source: Sci Rep. 2015 Sep 2;5:13684. doi: 10.1038/srep13684 (PMC4556958; doi:10.1038/srep13684)
Supplement: Supplementary Information [file srep13684-s1.pdf]

Combinatorial Pharmacophore Modeling of Multidrug and Toxin Extrusion Transporter 1 Inhibitors: a Theoretical Perspective for Understanding Multiple Inhibitory Mechanisms

Yuan Xu[†], *Xian Liu*[†], *Yulan Wang*[†], *Nannan Zhou*[†], *Jianlong Peng*[†], *Likun Gong*[‡,*], *Jing Ren*[‡], *Cheng Luo*[†], *Xiaomin Luo*[†,*], *Hualiang Jiang*[†,§], *Kaixian Chen*[†,§], *Mingyue Zheng*[†,*]

[†]State Key Laboratory of Drug Research, Shanghai Institute of Materia Medica, Chinese Academy of Sciences, 555 Zuchongzhi Road, Shanghai 201203, China

[‡]Center for drug safety evaluation and research, Shanghai Institute of Materia Medica, Chinese Academy of Sciences, 555 Zuchongzhi Road, Shanghai 201203, China

[§]School of Life Science and Technology, Shanghai Tech University, Shanghai 200031, China

[*]Correspondence should be made to: Likun Gong (lkgong@cdser.simm.ac.cn), Xiaomin Luo (xmluo@simm.ac.cn) and Mingyue Zheng (myzheng@simm.ac.cn)

Supplementary Table S1. Candidate pharmacophore hypotheses used for constructing CP model.

In.	Hypothesis	Survival score	Survival-inactive score	Selectivity score	Train_in	Train_non	Test_in	Test_non
**1**	HHR1*	2.88	2.68	1.10	10	47	12	58
**2**	HHR2*	2.95	2.89	0.97	8	15	7	21
**3**	HHR3	2.79	2.22	1.54	8	26	3	20
**4**	HHR4	3.01	2.89	1.01	11	31	9	28
**5**	HHH	2.00	1.91	0.88	9	42	7	50
**6**	DRR*	2.74	2.60	1.02	11	33	11	36
**7**	DHR1	2.71	2.52	1.12	9	51	9	43
**8**	DHR2	2.88	2.18	1.04	9	16	7	16
**9**	ARR	3.07	2.99	1.00	10	20	11	32
**10**	APR	2.87	2.74	1.25	11	31	14	26
**11**	AHP	2.92	2.85	1.11	7	15	5	13
**12**	AHH	2.96	2.78	1.09	6	42	8	54
**13**	ADR	2.92	2.80	1.04	12	35	13	24
**14**	ADH1	2.91	2.77	1.09	12	35	8	36
**15**	ADH2	2.89	2.80	1.07	10	25	5	23
**16**	AAR	2.81	2.68	0.94	11	35	12	36
**17**	AAP	2.99	2.91	1.31	8	15	5	16
**18**	HHHR	2.59	2.19	1.49	7	21	2	23
**19**	DHHR	2.33	1.72	1.55	6	22	6	35
**20**	DDHR	2.37	1.75	1.48	7	41	5	33
**21**	AHPR	3.00	2.74	1.65	7	8	5	8
**22**	AHHR1	2.71	1.99	1.49	7	33	6	35
**23**	AHHR2	2.63	1.97	1.50	7	32	6	36
**24**	AAHP	2.98	2.27	1.56	11	21	7	21
**25**	AAAP1*	3.06	2.42	1.62	11	22	7	24
**26**	AAAP2	3.07	2.41	1.59	9	23	7	21
**27**	AAHHR	2.43	1.41	1.74	8	37	7	31

Train_in: The number of matched inhibitors in training set.
Train_non: The number of matched noninhibitors in training set.
Test_in: The number of matched inhibitors in test set.
Test_non: The number of matched noninhibitors in test set.
*The hypotheses consist of the final CP model.

Supplementary Table S2. The site distances of single pharmacophore hypotheses comprising the obtained CP model.

Hypothesis	Site1	Site2	Distance(Å)
**HHR1**	H2	H1*	2.918
	H2	R1	3.789
	H1	R1	3.240
**DRR**	D1	R1	8.614
	D1	R2	3.241
	R1	R2	7.251
**HHR2**	H2	H1	6.348
	H2	R1	9.566
	H1	R1	13.153
**AAAP**	A1	A2	4.575
	A1	A3	4.228
	A1	P1	8.994
	A2	A3	5.347
	A2	P1	11.229
	A3	P1	7.517

*The feature numbers are used to distinguish the same type of features in the same hypothesis.

Supplementary Table S3. The site angles of single pharmacophore hypotheses comprising the obtained CP model.

**Hypothesis**	**Site1**	**Site2**	**Site3**	**Angle(degree)**
**HHR1**	H1*	H2	R1	56.0
	H2	H1	R1	75.7
	H2	R1	H1	48.3
**DRR**	R1	D1	R2	54.9
	D1	R1	R2	21.4
	D1	R2	R1	103.7
**HHR2**	H1	H2	R1	109.8
	H2	H1	R1	43.2
	H2	R1	H1	27.0
**AAAP**	A2	A1	A3	74.7
	A2	A1	P1	107.2
	A4	A1	P1	56.2
	A1	A2	A3	49.7
	A1	A2	P1	49.9
	A3	A2	P1	35.2
	A1	A3	A2	55.6
	A1	A3	P1	95.9
	A2	A3	P1	120.7
	A1	P1	A2	22.9
	A1	P1	A3	27.9
	A2	P1	A3	24.2

*The feature numbers are used to distinguish the same type of features in the same hypothesis.

Supplementary Table S4. The spatial arrangements of HHR4 in OCT2 CP model [1].

Hypothesis	Site1	Site2	Distance (Å)
**HHR4**	H1*	H2	5.78
	H1	R	2.95
	H2	R	3.89

*The feature numbers are used to distinguish the same type of features in the same hypothesis.

Supplementary Table S5. The list of all compounds used in this study

No	Name	Inhibitor (Yes/No)	PubChem ID
1	1-(2-Chlorobenzoyl)-4-(Phenylmethyl)-Piperidine	Y	667590
2	Alpha-ERGOCRYPTINE	Y	99049
3	Amiloride	Y	16231
4	Amlodipine	Y	2162
5	Antimycin A	Y	12550
6	Azelastine	Y	2267
7	Bazedoxifene	Y	154257
8	Benzethonium Chloride	Y	8478
9	Bexarotene	Y	82146
10	Bithionol	Y	2406
11	Buspirone	Y	2477
12	Caffeic Acid Phenethyl Ester	Y	108042
13	Camostat	Y	2536
14	Cetylpyridinium Bromide	Y	8816
15	Chlorhexidine	Y	62517
16	Ciclopirox	Y	2749
17	Cimetidine	Y	2756
18	Closantel	Y	42574
19	Danazol	Y	28417
20	Dihydroergocristine Mesylate	Y	444034
21	Dihydroergotamine	Y	3066
22	Domperidone	Y	3151
23	Doxazosin	Y	3157
24	Droperidol	Y	3168
25	D-Tubocurarine Chloride	Y	6000
26	Epinastine	Y	3241
27	Ergocornine	Y	3067
28	Ergonovine	Y	443884
29	Ergotamine	Y	3251
30	Ethinylestradiol	Y	5991
31	Famotidine	Y	3325
32	Fenbendazole	Y	3334
33	Gabexate	Y	3447
34	Genistein	Y	5280961
35	Gentian Violet	Y	3468
36	Granisetron	Y	3510
37	Guanfacine	Y	3519
38	Hexachlorophene	Y	3598
39	Idebenone	Y	3686
40	Imatinib	Y	5291
41	Indinavir	Y	60944
42	Kitasamycin	Y	5282189
43	L-744832	Y	6451153
44	Lamotrigine	Y	3878
45	Loperamide	Y	3955
46	Maprotiline	Y	4011
47	Mechlorethamine	Y	4033

(Continued)

48	Medroxyprogesterone Acetate	Y	6279
49	Megestrol Acetate	Y	11683
50	Mestranol	Y	6291
51	Metergoline	Y	28693
52	Methylene Blue	Y	4139
53	Mitoxantrone	Y	4212
54	Mosapride	Y	119584
55	Niacin	Y	938
56	Nifekalant	Y	4486
57	Norethindrone Acetate	Y	5832
58	Norgestrel	Y	13109
59	Ondansetron	Y	4595
60	Oxymetazoline	Y	4636
61	Oxytocin	Y	439302
62	Pantoprazole	Y	4679
63	Pentamidine	Y	4735
64	Perospirone	Y	115368
65	Phenacetin	Y	4754
66	Podophyllotoxin	Y	10607
67	Prazosin	Y	4893
68	Propoxyphene	Y	10100
69	Rimantadine	Y	5071
70	Ritonavir	Y	392622
71	Rwj-68354	Y	9819053
72	Salicylic Acid	Y	338
73	Selegiline	Y	5195
74	Sildenafil	Y	5212
75	Sulfacetamide	Y	5320
76	Sulindac Sulfide	Y	5352624
77	Tannic Acid	Y	250395
78	Terconazole	Y	441383
79	Thiabendazole	Y	5430
80	Topotecan	Y	60700
81	Trazodone	Y	5533
82	Tropisetron	Y	656665
83	Vecuronium Bromide	Y	39764
84	Zafirlukast	Y	5717
85	(-)-Thalidomide	N	92142
86	(+)-Thalidomide	N	75792
87	(R)-Bicalutamide	N	2375
88	(S)-(-)-Pindolol	N	688095
89	1-(2-Fluorobenzoyl)-4-(Phenylmethyl)-Piperidine	N	667749
90	1-(2-Methoxybenzoyl)-4-(Phenylmethyl)-Piperidine	N	667750
91	1-(2-Methylbenzoyl)-4-(Phenylmethyl)-Piperidine	N	1379258
92	1,3-Diphenylguanidine	N	7594
93	10,11-Dihydro-Carbamazepine	N	19099
94	17-Methyltestosterone	N	6010
95	1-Aminobenzotriazole	N	1367
96	1-Benzylimidazole	N	77918

(Continued)

97	1-Naphthyl Isothiocyanate	N	11080
98	2-Acetylaminofluorene	N	5897
99	2-HYDROXYPROPYL-Beta-CYCLODEXTRIN	N	44134771
100	2-Mercaptobenzothiazole	N	697993
101	3,3',4',5-Tetrachlorosalicylanilide	N	14385
102	3-Acetamidophenol	N	12124
103	3-Aminobenzamide	N	1645
104	4-(Phenylmethyl)-1-[2-(Trifluoromethyl)Benzoyl]-Piperidine	N	17683252
105	4,4'-Diethylaminoethoxyhexestrol	N	66143
106	4-Amino-1,8-Naphthalimide	N	1720
107	4-Methylpyrazole	N	3406
108	4-Nitrobenzoic Acid	N	6108
109	4-Nonylphenol	N	1752
110	4-Octylphenol	N	15730
111	5-Fluoro-2'-Deoxyuridine	N	5790
112	6(5h)-Phenanthridinone	N	1853
113	6-Mercaptopurine	N	667490
114	6-Methoxy-2-Naphthylacetic Acid	N	32176
115	8-Azaguanine	N	8646
116	Acarbose	N	41774
117	Aceclofenac	N	71771
118	Acemetacin	N	1981
119	Acetaminophen	N	1983
120	Acetazolamide	N	1986
121	Acetylcholine Chloride	N	6060
122	Acetylcysteine	N	12035
123	Aclarubicin	N	2008
124	Aconitine	N	2012
125	Actarit	N	2018
126	Acyclovir	N	2022
127	Adapalene	N	60164
128	Adrenosterone	N	223997
129	Albendazole	N	2082
130	Alfacalcidol	N	2091
131	Allopurinol	N	2094
132	Alpha-CYCLODEXTRIN	N	24796
133	Alpha-CYPERMETHRIN	N	49833
134	Alpha-NAPHTHOFLAVONE	N	11790
135	Alprazolam	N	2118
136	Alprenolol	N	2119
137	Alprostadil	N	5280723
138	Aluminum Lactate	N	92422
139	Amantadine	N	2130
140	Ambroxol	N	2132
141	Aminacrine	N	7019
142	Amineptine	N	34870
143	Aminoglutethimide	N	2145
144	Aminopterin	N	169371
145	Aminosalicylic Acid	N	4649

(Continued)

146	Amiodarone	N	2157
147	Amitraz	N	36324
148	Amitriptyline	N	2160
149	Amorolfine	N	2168
150	Amphotericin B	N	14956
151	Ampiroxicam	N	2176
152	Amprenavir	N	44342222
153	Amrinone	N	3698
154	Amsulosin	N	121829
155	Amygdalin(D)	N	2180
156	Anastrozole	N	2187
157	Androsterone	N	5879
158	Aniracetam	N	2196
159	Anisindione	N	2197
160	Antazoline	N	2200
161	Antipyrine	N	2206
162	Artemether	N	68911
163	Artemisinin	N	68827
164	Ascorbic Acid	N	54670067
165	Aspirin	N	2244
166	Astemizole	N	2247
167	Atenolol	N	2249
168	Atractyloside	N	2255
169	Atropine	N	3661
170	Auranofin	N	6918453
171	Azacitidine	N	9444
172	Azaribine	N	16574
173	Azaserine	N	830
174	Azathioprine	N	2265
175	Azauridine	N	5901
176	Azelaic Acid	N	2266
177	Azithromycin	N	55185
178	Azlocillin	N	2271
179	Aztreonam	N	2274
180	Bacitracin	N	2283
181	Balsalazide	N	5362070
182	Bay 11-7085	N	6603741
183	Beclomethasone	N	20469
184	Beclomethasone Dipropionate	N	21700
185	Bemegride	N	2310
186	Benazepril	N	5362124
187	Bendazac	N	2313
188	Bendroflumethiazide	N	2315
189	Benoxaprofen	N	39941
190	Benoxinate	N	4633
191	Benserazide	N	2327
192	Benzo[A]Pyrene	N	2336
193	Benzo[E]Pyrene	N	9128
194	Benzoic Acid	N	243

(Continued)

195	Benztropine	N	441353
196	Benzyl Alcohol	N	244
197	Beta Cyclodextrin	N	444294
198	Beta-ESTRADIOL	N	5757
199	Beta-ESTRADIOL 3-BENZOATE	N	222757
200	Betahistine	N	2366
201	Betaine	N	247
202	Betamethasone	N	9782
203	Betamipron	N	71651
204	Beta-NAPHTHOFLAVONE	N	2361
205	Bezafibrate	N	39042
206	Bifonazole	N	2378
207	Bisacodyl	N	2391
208	Bisphenol A	N	6623
209	Bopindolol	N	44112
210	Boric Acid	N	7628
211	Bretylium Tosylate	N	2431
212	Brilliant Green	N	12450
213	Brimonidine	N	2435
214	Bromfenac	N	60726
215	Bromhexine	N	2442
216	Bromisovalum	N	1176
217	Brompheniramine	N	6834
218	Budesonide	N	5281004
219	Bufexamac	N	2466
220	Buflomedil	N	2467
221	Buformin	N	2468
222	Bupivacaine Hydrochloride	N	64737
223	Bupropion	N	444
224	Busulfan	N	2478
225	Butamben	N	2482
226	Butenafine	N	2484
227	Butylparaben	N	7184
228	C8 Ceramide	N	5702614
229	Cadmium Acetate	N	10986
230	Caffeine	N	2519
231	Calcitriol	N	2524
232	Candesartan	N	2541
233	Cantharidin	N	2545
234	Capsaicin	N	2548
235	Captopril	N	2550
236	Carbachol	N	2551
237	Carbamazepine	N	2554
238	Carbimazole	N	31072
239	Carbinoxamine	N	2564
240	Carisoprodol	N	2576
241	Carmofur	N	2577
242	Carmustine	N	2578
243	Carvedilol	N	2585

(Continued)

244	Cefdinir	N	6398970
245	Cefixime	N	2675
246	Cefmetazole	N	2626
247	Cefotaxime	N	2632
248	Cefpodoxime Proxetil	N	441393
249	Cefuroxime	N	2659
250	Celecoxib	N	2662
251	Cerivastatin	N	2676
252	Cetirizine	N	2678
253	Cetraxate	N	2680
254	Chlordiazepoxide	N	2712
255	Chlormadinone Acetate	N	9324
256	Chlorobutanol	N	5977
257	Chlorothiazide	N	2720
258	Chloroxylenol	N	2723
259	Chlorphenesin Carbamate	N	2724
260	Chlorpheniramine	N	2725
261	Chlorpromazine	N	2726
262	Chlorpropamide	N	2727
263	Chlorprothixene	N	2729
264	Chlorquinaldol	N	6301
265	Chlorzoxazone	N	2733
266	Cholecalciferol	N	5283711
267	Cholic Acid	N	221493
268	Choline Chloride	N	6209
269	Cilostazol	N	2754
270	Cinnamaldehyde	N	307
271	Cinnarizine	N	2761
272	Cisapride	N	2769
273	Citalopram	N	2771
274	Citric Acid	N	311
275	Cladribine	N	1546
276	Clarithromycin	N	54688
277	Clebopride	N	2780
278	Clenbuterol	N	2783
279	Clidinium Bromide	N	19004
280	Clioquinol	N	2788
281	Clobetasol Propionate	N	32798
282	Clofibric Acid	N	2797
283	Clomacran	N	21382
284	Clomiphene	N	60974
285	Clomipramine	N	2801
286	Clonazepam	N	2802
287	Clonidine	N	2803
288	Clopidogrel	N	2806
289	Clorprenaline	N	2810
290	Clotrimazole	N	2812
291	Cloxacillin	N	6098
292	Clozapine	N	2818

(Continued)

293	Cobalt(Ii) Acetylacetonate	N	407383
294	Cocculin	N	5311359
295	Colchicine	N	6167
296	Colistin	N	73091
297	Cortisone	N	5753
298	Coumarin	N	323
299	Cromolyn	N	2882
300	Crotamiton	N	6604255
301	Cyclandelate	N	2893
302	Cyclobenzaprine	N	2895
303	Cycloheximide	N	6197
304	Cyclophosphamide	N	2907
305	Cyproheptadine	N	2913
306	Cyproterone Acetate	N	9880
307	Cyromazine	N	47866
308	Cytarabine	N	6253
309	Dacarbazine	N	5281007
310	Dactinomycin	N	2019
311	Dapiprazole	N	3033538
312	Dapsone	N	2955
313	Daunorubicin	N	30323
314	D-Camphor	N	159055
315	Deferoxamine	N	2973
316	Dehydrocholic Acid	N	6674
317	Dequalinium Chloride	N	2993
318	Desipramine	N	2995
319	Deslanoside	N	2996
320	Desloratadine	N	124087
321	Desoximetasone	N	5311067
322	Dexamethasone	N	5743
323	Dexamethasone Phosphate	N	9400
324	Dexbrompheniramine	N	16960
325	Dexchlorpheniramine	N	33036
326	Dexfenfluramine	N	65801
327	Dexibuprofen	N	39912
328	Dexketoprofen	N	667550
329	Dexpanthenol	N	131204
330	Dextromethorphan	N	5362449
331	D-Galactosamine	N	2794221
332	Diazepam	N	3016
333	Diazoxide	N	3019
334	Dibucaine	N	3025
335	Dichlorvos	N	3039
336	Diclofenac	N	3033
337	Dicyclomine	N	3042
338	Didanosine	N	3043
339	Dideoxycytidine	N	24066
340	Dieldrin	N	969491
341	Diethylcarbamazine	N	3052

(Continued)

342	Diethylstilbestrol	N	448537
343	Diflorasone Diacetate	N	71414
344	Diflunisal	N	3059
345	Digitonin	N	25444
346	Digitoxin	N	3061
347	Digoxin	N	3062
348	Diltiazem	N	39186
349	Dimenhydrinate	N	10660
350	Diphenhydramine	N	3100
351	Diphenidol	N	3055
352	Dipyridamole	N	3108
353	Disopyramide	N	3114
354	Disulfiram	N	3117
355	Dobutamine	N	36811
356	Docetaxel	N	64780
357	Donepezil	N	3152
358	Doxapram	N	3156
359	Doxepin	N	667477
360	Doxifluridine	N	3159
361	Doxofylline	N	50942
362	Doxorubicin	N	31703
363	Doxycycline	N	373075
364	Dyclonine Hydrochloride	N	3180
365	Dyphylline	N	3182
366	Ebastine	N	3191
367	Econazole	N	3198
368	Edaravone	N	4021
369	Edrophonium Chloride	N	8307
370	Efavirenz	N	3203
371	Emetine	N	10219
372	Enalapril	N	5362032
373	Enrofloxacin	N	71188
374	Epalrestat	N	1549120
375	Eperisone	N	3236
376	Epirubicin	N	41867
377	Erythromycin	N	3255
378	Erythromycin Propionate	N	3258
379	Esmolol	N	59768
380	Estriol	N	5756
381	Estrone	N	5870
382	Ethacrynic Acid	N	3278
383	Ethambutol	N	3279
384	Ethionamide	N	3284
385	Ethisterone	N	5284557
386	Ethosuximide	N	3291
387	Ethylenediamine	N	3301
388	Ethylestrenol	N	13765
389	Ethylparaben	N	8434
390	Ethynodiol Diacetate	N	9270

(Continued)

391	Etodolac	N	3308
392	Etoposide	N	3310
393	Eucalyptol (Cineole)	N	2758
394	Exemestane	N	60198
395	Felbinac	N	3332
396	Felodipine	N	3333
397	Fenbufen	N	3335
398	Fenofibrate	N	3339
399	Fenoprofen	N	3342
400	Ferulic Acid	N	709
401	Fexofenadine	N	3348
402	Finasteride	N	57363
403	Fluconazole	N	3365
404	Flucytosine	N	3366
405	Fludarabine	N	30751
406	Fludrocortisone Acetate	N	225609
407	Flufenamic Acid	N	3371
408	Flumazenil	N	3373
409	Flunarizine	N	941361
410	Flunisolide	N	82153
411	Flunoxaprofen	N	39940
412	Fluocinolone Acetonide	N	6215
413	Fluocinonide	N	9642
414	Fluorometholone	N	9878
415	Fluorouracil	N	3385
416	Fluoxetine	N	3386
417	Fluphenazine	N	3372
418	Flurandrenolide	N	15209
419	Flurbiprofen	N	3394
420	Folic Acid	N	3405
421	Formoterol	N	3410
422	Furazolidone	N	5323714
423	Furosemide	N	3440
424	Galantamine	N	3449
425	Gallamine Triethiodide	N	3450
426	Gamma-Cyclodextrin	N	86575
427	Ganciclovir	N	3454
428	Gemcitabine	N	3461
429	Gemfibrozil	N	3463
430	Genaconazole (RR(+) Isomer)	N	60741
431	Geraniol	N	637566
432	Gliclazide	N	3475
433	Glimepiride	N	3476
434	Glipizide	N	3478
435	Glyburide	N	3488
436	Glycolic Acid	N	757
437	Glycyrrhizin	N	14982
438	Goserelin	N	47725
439	Gramicidin	N	73357

(Continued)

440	Griseofulvin	N	3512
441	Guaifenesin	N	3516
442	Guanabenz	N	3517
443	GW-1929	N	6518171
444	Halcinonide	N	443943
445	Haloperidol	N	3559
446	Haloprogin	N	3561
447	Homatropine Hydrobromide	N	5821
448	Homochlorcyclizine	N	3627
449	Huperzine A	N	1253
450	Hydralazine	N	3637
451	Hydrazine	N	9321
452	Hydrochlorothiazide	N	3639
453	Hydrocortisone	N	5754
454	Hydroflumethiazide	N	3647
455	Hydroxyprogesterone	N	6238
456	Hydroxyurea	N	3657
457	Hydroxyzine	N	3658
458	Hymecromone	N	5280567
459	Hyoscyamine	N	64692
460	Ibudilast	N	3671
461	Ibuprofen	N	3672
462	Icodextrin	N	79025
463	Idarubicin	N	42593
464	Idoxuridine	N	3687
465	Ifosfamide	N	3690
466	Imipramine	N	3696
467	Indapamide	N	3702
468	Indomethacin	N	3715
469	Inosine	N	804
470	Inositol	N	892
471	Iodoquinol	N	3728
472	Ipratropium Bromide	N	31098
473	Ipriflavone	N	3747
474	Iproniazid	N	3748
475	Irbesartan	N	3749
476	Irinotecan	N	3750
477	Irsogladine Maleate	N	5282435
478	Isoeugenol	N	1549041
479	Isoniazid	N	3767
480	Isoprenaline	N	3779
481	Isopropamide Iodide	N	6284
482	Isotretinoin	N	5282379
483	Itopride	N	3792
484	Ivermectin	N	3085416
485	Kainic Acid	N	3816
486	Ketamine	N	3821
487	Ketoprofen	N	3825
488	Ketorolac	N	3826

(Continued)

489	Ketotifen	N	3827
490	Labetalol	N	3869
491	Lacidipine	N	68733
492	Lactulose	N	3872
493	Lafutidine	N	3873
494	Lamivudine	N	3877
495	Lansoprazole	N	3883
496	L-Camphor	N	444795
497	Leflunomide	N	3899
498	Letrozole	N	3902
499	Leuprolide	N	3911
500	Levobunolol	N	39468
501	Levobupivacaine	N	2474
502	Levodopa	N	6047
503	Levofloxacin	N	149096
504	Lidocaine	N	3676
505	Lincomycin	N	3928
506	Linezolid	N	3929
507	Lisuride	N	28864
508	Lomerizine	N	3949
509	Lomustine	N	3950
510	Loratadine	N	3957
511	Lorazepam	N	3958
512	Losartan	N	3961
513	Lovastatin	N	3962
514	L-Phenylephrine	N	6041
515	Lysergol	N	3982
516	Mannitol	N	453
517	Mc 258714	N	3385213
518	Mc 258717	N	3421241
519	Mc 288769	N	1378765
520	Mebeverine	N	4031
521	Meclizine	N	4034
522	Meclofenamic Acid	N	4037
523	Meclofenoxate	N	4039
524	Medroxyprogesterone	N	10631
525	Mefenamic Acid	N	4044
526	Melatonin	N	896
527	Menatetrenone	N	4056
528	Mepazine	N	6075
529	Mepenzolate Bromide	N	4057
530	Mepivacaine	N	4062
531	Meropenem	N	441130
532	Mesalamine	N	4075
533	Mesna	N	598
534	Mestanolone	N	10633
535	Metaraminol	N	4087
536	Metformin	N	4091
537	Methapyrilene	N	4098

(Continued)

538	Methazolamide	N	4100
539	Methimazole	N	1349907
540	Methocarbamol	N	4107
541	Methotrexate	N	126941
542	Methoxamine	N	6082
543	Methoxsalen (Xanthtoxin)	N	4114
544	Methscopolamine Bromide	N	5459110
545	Methychlothiazide	N	4121
546	Methyl Salicylate	N	4133
547	Methylergonovine	N	4140
548	Methylparaben	N	7456
549	Metoclopramide	N	4168
550	Metolazone	N	4170
551	Metoprolol	N	4171
552	Metrifonate	N	5853
553	Metyrapone	N	4174
554	Mevastatin	N	2854
555	Mexiletine	N	4178
556	Mianserin	N	4184
557	Miconazole	N	4189
558	Midecamycin	N	290482
559	Midodrine	N	4195
560	Mifepristone	N	55245
561	Milrinone	N	4197
562	Minaprine	N	4199
563	Minoxidil	N	4201
564	Mirtazapine	N	4205
565	Misoprostol	N	4183806
566	Mitotane	N	4211
567	Moclobemide	N	4235
568	Modafinil	N	4236
569	Mometasone Furoate	N	55188
570	Monobenzone	N	7638
571	Monocrotaline	N	9415
572	Montelukast	N	5281040
573	Moxonidine	N	4810
574	Mycophenolate Mofetil	N	5281078
575	Myrtecaine	N	71851
576	N,N'-Diphenyl-P-Phenylenediamine	N	6319
577	Nabumetone	N	4409
578	Nadolol	N	4411
579	Nafenopin	N	19592
580	Naftopidil	N	4418
581	Nalbuphine	N	5311304
582	Nalidixic Acid	N	4421
583	Naloxonazine	N	4424
584	Naloxone	N	5284596
585	Nandrolone	N	9904
586	Naphazoline	N	4436

(Continued)

587	Naproxen	N	1302
588	Nateglinide	N	4443
589	Nefazodone	N	4449
590	Nelfinavir	N	4451
591	Neostigmine Bromide	N	4456
592	Niacinamide	N	936
593	Nicardipine	N	4474
594	Nifedipine	N	4485
595	Niflumic Acid	N	4488
596	Nifursol	N	28140
597	Nilutamide	N	4493
598	Nimesulide	N	4495
599	Nimetazepam	N	4496
600	Nimodipine	N	4497
601	Nisoldipine	N	4499
602	Nitrazepam	N	4506
603	Nitrendipine	N	4507
604	Nitrofurantoin	N	4509
605	Nitrofurazone	N	1839
606	Nizatidine	N	4513
607	Nocodazole	N	4122
608	Norepinephrine	N	951
609	Norethindrone	N	6230
610	Norfloxacin	N	4539
611	Nortriptyline	N	4543
612	Noscapine	N	4544
613	Novartis P-38 Inhibitor	N	9819930
614	Ns-398	N	4553
615	Nylidrin	N	4567
616	Nystatin	N	3468412
617	Octreotide	N	54374
618	Olanzapine	N	4585
619	Omeprazole	N	4594
620	Orlistat	N	4599
621	Orphenadrine	N	4601
622	Ouabain	N	439501
623	Oxaliplatin	N	43805
624	Oxaprozin	N	4614
625	Oxatomide	N	4615
626	Oxcarbazepine	N	34312
627	Oxfendazole	N	40854
628	Oxiconazole	N	4623
629	Oxybenzone	N	4632
630	Oxybutynin	N	4634
631	Oxymetholone	N	3032303
632	Oxyquinoline	N	1923
633	Pancuronium Bromide	N	27350
634	Panthenol	N	4678
635	Pantothenic Acid	N	988

(Continued)

636	Paroxetine	N	4691
637	Parthenolide	N	927704
638	Pemoline	N	4723
639	Penciclovir	N	4725
640	Penicillic Acid	N	304729
641	Penicillin G	N	2349
642	Pentoxifylline	N	4740
643	Perflubron	N	9873
644	Perflunafene	N	9386
645	Pergolide	N	4745
646	Perhexiline	N	4746
647	Perphenazine	N	4748
648	Phenacemide	N	4753
649	Phenazopyridine	N	4756
650	Phenelzine	N	3675
651	Phenformin	N	8249
652	Phenindione	N	4760
653	Pheniramine	N	4761
654	Phenobarbital	N	4763
655	Phenol	N	996
656	Phenothiazine	N	7108
657	Phenoxybenzamine	N	4768
658	Phenprobamate	N	4770
659	Phentolamine	N	5775
660	Phenyl Salicylate	N	8361
661	Phenylbutazone	N	4781
662	Phenylhydrazine	N	7516
663	Phenylpropanolamine	N	4786
664	Phenyltoloxamine	N	7077
665	Physostigmine	N	4811
666	Piclamilast	N	154575
667	Pilocarpine	N	5910
668	Pindolol	N	4828
669	Pioglitazone	N	4829
670	Piperacillin	N	31864
671	Piperilate Ethobromide	N	168088
672	Piracetam	N	4843
673	Polymyxin B1	N	199402
674	P-Phenylenediamine	N	7814
675	Pralidoxime Chloride	N	5353894
676	Pramoxine	N	4886
677	Pravastatin	N	4889
678	Praziquantel	N	4891
679	Prednisolone	N	5755
680	Prednisone	N	5865
681	Pridinol	N	4904
682	Prilocaine	N	4906
683	Primaquine	N	4908
684	Primidone	N	44909

(Continued)

685	Prinomastat	N	466151
686	Probenecid	N	4911
687	Probucol	N	4912
688	Procainamide	N	4913
689	Procaine	N	4914
690	Procarbazine	N	4915
691	Prochlorperazine	N	4917
692	Procyclidine	N	4919
693	Proglumide	N	4922
694	Promazine	N	4926
695	Promethazine	N	4927
696	Propafenone	N	4932
697	Propantheline Bromide	N	4934
698	Proparacaine	N	4935
699	Propofol	N	4943
700	Propranolol	N	4946
701	Propylene Glycol	N	1030
702	Propylparaben	N	7175
703	Propylthiouracil	N	657298
704	Proscillaridin	N	5284613
705	Pseudoephedrine	N	7028
706	Puromycin	N	4984
707	Pyrazinamide	N	1046
708	Pyridoxine	N	1054
709	Pyrilamine	N	4992
710	Pyrithione Zinc	N	26041
711	Pyrogallol	N	1057
712	Quetiapine	N	5002
713	Quinapril	N	54892
714	Quinidine	N	441074
715	Quinine	N	3034034
716	R(-)-Apomorphine	N	6005
717	R(-)-Ibuprofen	N	114864
718	R-(+)-Propranolol	N	21138
719	Rabeprazole	N	5029
720	Racepinephrine	N	838
721	Raloxifene	N	5035
722	Ramipril	N	5362129
723	Ranitidine	N	5039
724	Rebamipide	N	5042
725	Resorcinol	N	5054
726	Resveratrol	N	445154
727	Ribavirin	N	5064
728	Riboflavin	N	710
729	Rifabutin	N	5361912
730	Rifampin	N	5381226
731	Rifamycin B	N	26340
732	Rifapentine	N	6323497
733	Riluzole	N	5070

(Continued)

734	Roflumilast	N	449193
735	Rolipram	N	5092
736	Ropivacaine	N	71273
737	Rotenone	N	5102
738	Roxithromycin	N	5106
739	Rubitecan	N	65567
740	S(-)-Carbidopa	N	34359
741	Safrole	N	5144
742	Salicylamide	N	5147
743	Salinomycin	N	5151
744	Salsalate	N	5161
745	Santoflex(R) Ip	N	7573
746	Saquinavir	N	60787
747	SCH-351591	N	9802841
748	Scopolamine	N	5184
749	Secnidazole	N	71815
750	Semustine	N	5198
751	Sertraline	N	5203
752	Sevoflurane	N	5206
753	Sibutramine	N	5210
754	Simvastatin	N	54454
755	Sorbinil	N	5252
756	Sorbinil Enatiomer	N	5748291
757	Sotalol	N	5253
758	Sparteine	N	644020
759	Spectinomycin	N	2021
760	Spiperone	N	5265
761	Spironolactone	N	6419955
762	Sporidesmin A	N	99596
763	Streptozotocin	N	5299
764	Strontium Chloride Hexahydrate	N	159250
765	Strychnine	N	441071
766	Succimer	N	9354
767	Sulbactam	N	5316
768	Sulconazole	N	5318
769	Sulfadiazine	N	5215
770	Sulfadimethoxine	N	5323
771	Sulfadoxine	N	17134
772	Sulfamerazine	N	5325
773	Sulfamethazine	N	5327
774	Sulfamethizole	N	5328
775	Sulfamethoxazole	N	5329
776	Sulfamonomethoxine	N	5332
777	Sulfanilamide	N	5333
778	Sulfaphenazole	N	5335
779	Sulfasalazine	N	5353980
780	Sulfathiazole	N	5340
781	Sulfinpyrazone	N	5342
782	Sulfisoxazole	N	5344

(Continued)

783	Sulindac	N	1548887
784	Sulindac Sulfone	N	114866
785	Sulpiride	N	5355
786	Sumatriptan	N	5358
787	Suxamethonium Chloride	N	5314
788	Tacrine	N	1935
789	Tamoxifen	N	2733526
790	Tazobactam	N	4836
791	Tegafur	N	5386
792	Telmisartan	N	65999
793	Tenidap	N	57946
794	Tenofovir Disoproxil	N	464205
795	Tenoxicam	N	54677971
796	Terazosin	N	5401
797	Terbinafine	N	5402
798	Terbutaline	N	5403
799	Terfenadine	N	5405
800	Tetracaine	N	5411
801	Tetracycline	N	54675776
802	Tetrahydrozoline	N	5419
803	Tetramethylthiuram Monosulfide	N	7347
804	Thalidomide	N	5426
805	Theophylline	N	2153
806	Thiamine	N	1130
807	Thiamphenicol	N	5433
808	Thimerosal	N	5908
809	Thioacetamide	N	2723949
810	Thioctic Acid	N	864
811	Thioguanine	N	2723601
812	Thioridazine	N	5452
813	Thiosalicylic Acid	N	5443
814	Thiotepa	N	5453
815	Thiram	N	5455
816	Thymol	N	6989
817	Tiapride	N	5467
818	Tibolone	N	444008
819	Ticlopidine	N	5472
820	Ticrynafen	N	38409
821	Timolol	N	5478
822	Tinidazole	N	5479
823	Tiopronin	N	5483
824	Tirilazad	N	60516
825	Tocainide	N	38945
826	Tolazamide	N	5503
827	Tolazoline	N	5504
828	Tolbutamide	N	5505
829	Tolfenamic Acid	N	5507
830	Tolnaftate	N	5510
831	Tolperisone	N	5511

(Continued)

832	Tolterodine Tartrate	N	60774
833	Topiramate	N	5514
834	Toremifene	N	5516
835	Torsemide	N	41781
836	Tramadol	N	5523
837	Tranilast	N	5527
838	Tranylcypromine	N	5530
839	Triacetin	N	5541
840	Triamcinolone	N	31307
841	Triamcinolone Acetonide	N	6436
842	Trichlormethiazide	N	5560
843	Trifluoperazine	N	5566
844	Trihexyphenidyl	N	5572
845	Trimetazidine	N	21109
846	Trimethadione	N	5576
847	Trimipramine	N	5584
848	Tripelennamine	N	5587
849	Tromethamine	N	6503
850	Tropicamide	N	5593
851	Troxipide	N	5597
852	U-78517f	N	9808655
853	Ubenimex	N	72172
854	Urethane	N	5641
855	Ursodeoxycholic Acid	N	31401
856	Valacyclovir	N	5647
857	Valdecoxib	N	119607
858	Valethamate Bromide	N	5648
859	Valpromide	N	71113
860	Valsartan	N	5650
861	Vancomycin	N	14969
862	Venlafaxine	N	5656
863	Verapamil	N	2520
864	Vidarabine	N	21704
865	Vincristine	N	5978
866	Vindesine	N	40839
867	Vinorelbine	N	5672
868	Vitamin E Succinate	N	20353
869	VX-745	N	196969
870	Xylometazoline	N	5709
871	Y-27632	N	5711
872	Yohimbine	N	2866
873	Zaleplon	N	5719
874	Zaprinast	N	5722
875	Zidovudine	N	5726
876	Zileuton	N	60490
877	Zinc Dibutyldithiocarbamate	N	8684
878	Zolmitriptan	N	5731
879	Zolpidem	N	5732
880	Zomepirac	N	5733
881	Zopiclone	N	5735

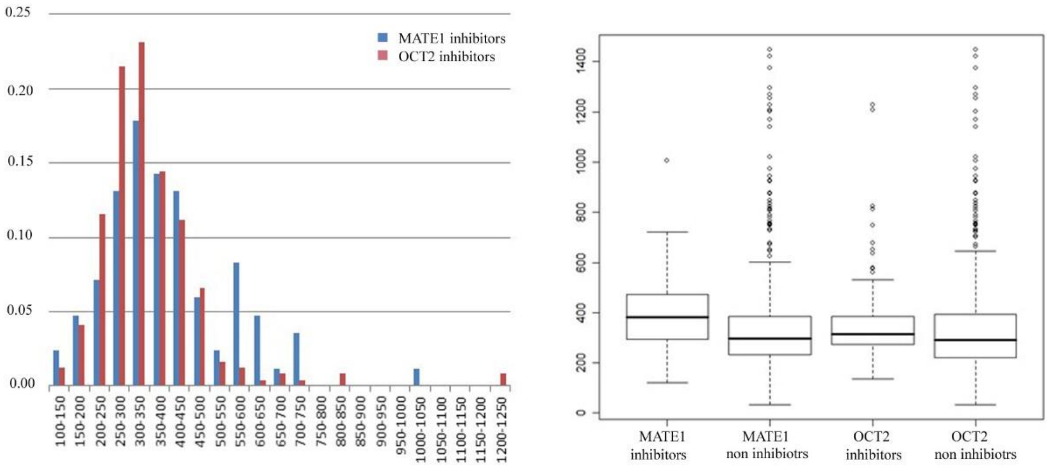

Supplementary Figure S1. The molecular weight distribution of MATE1 and OCT2 inhibitors.

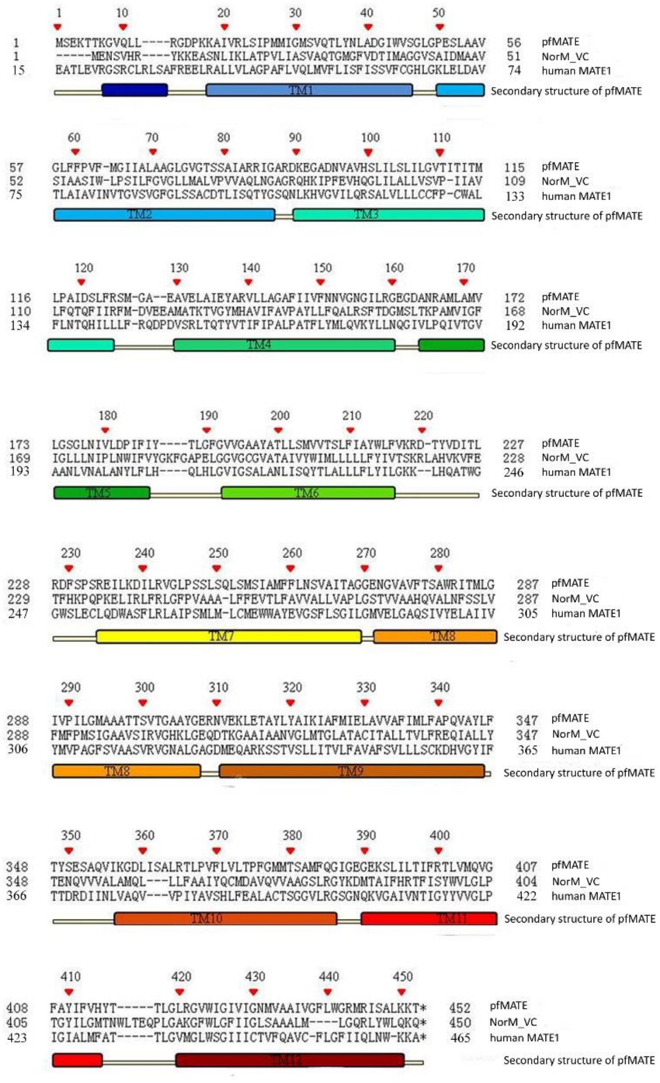

Supplementary Figure S2. Sequence alignment of MATE family transporters by MODELLER, including hMATE1, NorM_VC and pfMATE.

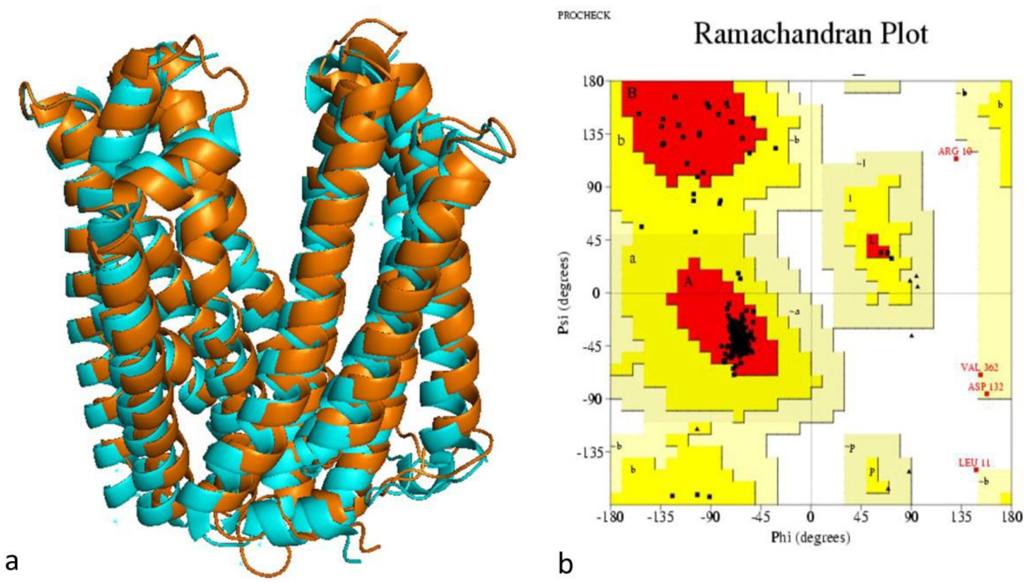

Supplementary Figure S3. The homology model of hMATE1.**a.**The structural alignment of homology model and pfMATE, where hMATE1 is in orange color, and the crystal structure of pfMATE in cyan. **b.** Ramachadran plot of homology model, where 95% residuals are in the core region.

References

1 Xu, Y. *et al.* Combinatorial Pharmacophore Modeling of Organic Cation Transporter 2 (OCT2) Inhibitors: Insights into Multiple Inhibitory Mechanisms. *Mol. Pharm.* **10**, 4611-4619 (2013).
